# Effect of the Buteyko breathing technique on asthma severity control among school age children

**DOI:** 10.1186/s43168-022-00149-3

**Published:** 2022-07-23

**Authors:** Esraa Elwan Mohammed Hassan, Fawzia Elsayed Abusaad, Boshra Attia Mohammed

**Affiliations:** Pediatric Nursing Department, Faculty of Nursing, Mansoura University, Mansoura, Egypt

**Keywords:** Asthma control, Breathing exercise, Buteyko breathing technique, Childhood asthma, School-age children

## Abstract

**Background:**

Asthma is a complex condition that can impair not only the child’s physical growth but also his optimal functional capacity and performance. Buteyko breathing technique is an exercise designed to regulate the breathing process. This study aimed to evaluate the effect of the Buteyko breathing technique on asthma severity control among school-age children. In Egypt, this technique was applied through five studies, four among adult patients and only one among children. In Mansoura University, only one study conducted among adult patients and no studies conducted among children. Therefore, to fulfill this gap of knowledge, it was necessary to study the effect of this technique on asthma severity control among school age children.

**Results:**

The mean childhood asthma control pretest was significantly improved in the posttest with high mean percent change of posttest than pretest (*p* = *0.0001*), which was clinically and statically high significant. There was a statistical significant increase in the mean of peak expiratory flow rate and control pause test at the fourth week than the first one (*p* = *0.0001*), with a high significant mean percent of change. There was a significant decrease in the heart rate over the 4 weeks of follow-up with high mean percent changes at fourth week than the first one (*p* = *0.003*).

**Conclusions:**

This study supports the effectiveness of the Buteyko breathing technique in improving respiratory outcome and promoting asthma control among school-age children with bronchial asthma.

**Trial registration:**

ClinicalTrials.gov, NCT05390554, registered on May 24 2022, retrospectively registered.

## Introduction

Life is dependent upon the action of breathing. Breathing is considered and believed as the most basic of all human body functions as it affects all body parts [1]. As defined by the [2], asthma is a disease that affects the lungs, which causes repeated episodes of wheezing, breathlessness, chest tightness, and nighttime or early morning coughing. It affects children in all age classes, but mostly begins in infancy. About 300 million people globally are affected by asthma, and it is likely that another 100 million will be affected by 2025 [3]. Over 80% of asthma-related deaths occur in low- and lower-middle income countries [4]. Asthma is a common health problem in Egypt and probably underdiagnosed and undertreated, particularly among children from poor families [5]. The overall prevalence of bronchial asthma in Egypt is 13.4% [6].

The pharmacological treatment of asthma includes reliever medications, which are drugs that allow relief of symptoms within few minutes, during worsening asthma or exacerbations, and controller medications, and that are used for maintenance treatment of asthma [7]. Despite greater understanding and novel public health and pharmacological measures that become available to reduce the prevalence of asthma, today, there is a worldwide public interest in physical therapy techniques and complementary alternative medicine (CAM) for asthma [3, 8].

The Buteyko breathing technique is one of CAM techniques that gains popularity. It was developed by Dr. Konstantin Buteyko in Russia in 1952. He believed persistent hidden over breathing (hyperventilation) to be a common, important, and generally unrecognized destabilizer of physiological processes and psychological states of asthmatic patients [9]. His theory is based on the discovered “Hyperventilation Syndrome” in 1871, which revealed that the heavy breathing in a calm state triggers dizziness and occasionally fainting. In addition to the discovered theory by Russian physiologists Verigo and Boher in 1904, who revealed that without CO_2_, O_2_ is bound to the hemoglobin in the blood and simply does not work effectively [10].

In Buteyko’s view, because CO_2_ was so important, the body developed a set of defensive mechanisms to maintain CO_2_, including constriction of airways and blood vessels, and giving rise to diseases such as asthma and hypertension. According to his theory, CO_2_ is a bronchodilator of the lung and low CO_2_ “hypocapnia” has exacerbated multiple medical problems and developed as many as 150 symptoms and conditions [10, 11]. Therefore, he believed that a small rise in the CO_2_ level has many beneficial results in the body including relaxing smooth muscle, increasing oxygenation, switching on the relaxing nervous system, and increasing the production of nitric oxide by the body. Relaxation of the bronchi and bronchioles can improve ventilation and greatly decrease airway spasms associated with asthma in the respiratory system [12].

Buteyko approach seeks to educate asthmatic patients to decrease airflow by teaching them the best way to hold their breath at the functional residual capacity [11]. The key component of the Buteyko program is to minimize hyperventilation by periods of controlled reduction of breathing, known as “slow breathing” and “reduced breathing,” coupled with periods of breath keeping, known as “control pauses” and “extended pauses” [13]. The use of the diaphragm for breathing is often recommended, and the use of accessory muscles for breathing is discouraged. They are sometimes accompanied in Buteyko by physical activities to increase the CO_2_ build-up [14].

Advice and instruction on the effects of nasal breathing over oral breathing are also used in the Buteyko technique. The nose not only warm, filter, and humidify the inspired air, but also creates nitric oxide, which is a strong bronchodilator for asthma. In order to encourage nasal breathing, Buteyko patients are encouraged to breathe through the nose during the day and try to tap the mouth at night. The Buteyko technique also proposes lifestyle changes beyond breathing, including diet, allergy avoidance, and stress control [14]. The Buteyko method’s four cardinal laws are as follows: keeping the mouth closed, keeping the back straight, breathing gently and silently, and eat only if hungry [15].

## Methods

### Research design

A quasi-experimental design of one group pre- and post-test was used to conduct this study.

### Study setting

The study was conducted at the outpatient clinics of Mansoura University Children Hospital (MUCH), which provides health services to children cases from Dakhlia governorate and Delta region. The sample was recruited from the sensitivity clinic that aims to follow up of children with bronchial asthma and children with various chest diseases.

### Subjects

The sample size cannot be calculated as there is no specific annual statistical report about the admitted asthma condition to sensitivity clinic, so a convenient sample of 33 asthmatic school-age children who fulfill the following criteria through the data collection period was recruited.

#### Inclusion criteria

- The school-age child of both sex whose age range from 6 to 12 years to insure the ability of the child to perform the Buteyko exercise.
- The child whose condition is mild to moderate as confirmed by the medical practitioner and the child’s hospital follow-up sheet.
- The child treated from asthma only by medications and no other alternative treatment as revealed by their primary caregiver.
- The child and their parents did not take any previous instruction about the Buteyko method.
- The child and their parents were accepting participation for 4 weeks during the study.

#### Exclusion criteria

- The child who does not have the inclusion criteria.
- The child with severe asthma, cardiac disease, mental retardation, pneumonia, infectious disease, lung disease, physical disabilities, and psychiatric disorders as confirmed by the medical practitioner.
- The child who receives any other alternative therapies.

**Fig. 1:**
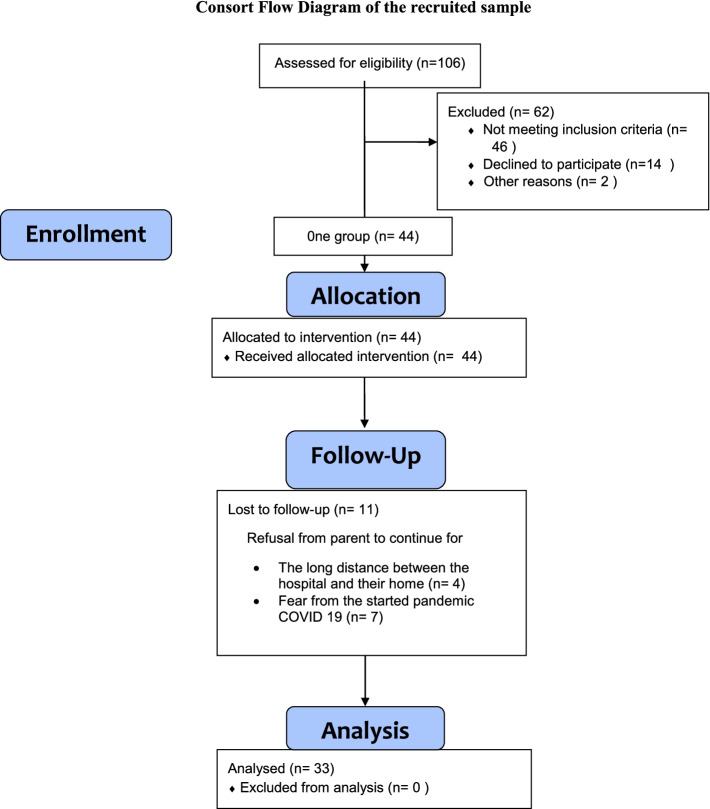
Consort flow diagram of the recruited sample

The sample size was limited because of the start of the COVID-19 pandemic which affects the children’s attendance to the clinic and refusal from mothers to continue in the study program (Fig. 1).

All cases that complete the course of exercise until the analysis phase did not acquire respiratory infection during this period as revealed by their parents and the clinical assessment.

### Ethical considerations

An ethical approval was obtained from the Research Ethics Committee. Then, the official permission was obtained from the hospital administrative authority to collect the data and conduct the study.

Participants and their parents were informed that the participation of this study is voluntary, and he\she has the right to accept or refuse participation of the study. Written informed consent was obtained from the children and their parents who accept participation of the study after providing them with a complete and detailed description of the study including the purposes and benefits. The participants were informed that the personal data will be kept confidential. All information taken from the participant was protected and does affect their status by any means and used only for the purpose of the research.

### Data collection tools

The data were collected by using the following three tools.

#### Tool one: Structured interview sheet for children and their mothers

It consisted of two parts as follows:

This part was developed by the researcher after reviewing relevant literatures [16, 17] and modifications of study supervisors. It is consisted with biodemographic and clinical data.

The tool was adapted from Liu et al. [18]. It is a valid test established by GlaxoSmithKline that involves seven questions to evaluate asthma control and the effects of asthma on diurnal activities among school age children. It has seven questions: the first four are answered by the children and the last three by their parents. Each question had four answer options for children and six answer options for parents. This part is completed at the first and final sessions as a pre- and post-test.

Its scores were categorized as the following according to Bime et al. [19]:

- 20:27 good asthma control
- 10:19 moderate asthma control
- Less than 10 poorly asthma control

#### Tool two: Peak expiratory flow rate (PEFR)

The peak expiratory flow rate (PEFR) is the person’s maximum speed of expiration. It is measured with a mini wright peak flow meter device to display the person’s capability to expire out air through the bronchi [20]. This test was done at the beginning, during follow-up test, and at the end of the sessions. According to [21], its interpretations are often categorized into three zones of depth: green, yellow, and red

- Green zone: The peak flow result is between 80 and 100% of the child’s predicted value.
- Yellow zone: Peak flow result is between 50 and 79% of child’s predicted value.
- Red zone: Peak flow result is less than 50% of the child’s predicted value.

#### Tool three: Control pause test (CP)

It was adopted from [22]. It was established by Dr. Buteyko to assess the depth of breathing and consequent retention of the carbon dioxide, resultant oxygenation, and health by using the special breathing holding manner. It was done at the beginning, during, and after the treatment program by using a stopwatch.

According to [23], the scoring was categorized as follows:

- Less than 10: health is severely affected.
- 10 to less than 20: patient probably suffering from a chronic illness, along with symptoms, such as blocked nose, snoring, insomnia, coughing, short breath, and asthma.
- 20 to less than 40: most symptoms are not there, but may occur following a triggering event.
- 40 and more: good health.

### *Study *intervention

It includes the preparatory phase and exploratory phase.

#### The preparatory phase

This phase included a review of the past and current-related literature and studies and develop the study tools. The learning booklet and story were prepared by the researcher in a simple Arabic language to meet the mother’s needs and motivate children after reviewing the articles and literature. The data collection instrument (children peak flow meter with number of disposable plastic mouthpieces, measuring tape, weight scale, stopwatch, disinfectant alcohol, and pen) was prepared by the researcher.

The content validity of the study tools was assessed and revised by a jury that involved a panel of five experts in the Faculty of Nursing Mansoura University, and modifications were made according to their opinions. The content reliability of the study tools was tested by using Cronbach’s alpha test as follows:

- Childhood asthma control test = 0.820
- Peak expiratory flow rate = 0.928
- Control pause test = 0.851

#### Exploratory phase

It includes pilot study and fieldwork.

- Pilot study
  A pilot study was carried out on 10% of the total sample (n = 4) to evaluate the clarity, feasibility, and applicability of the study tools, educational booklet, and story. This number was included to the study sample as there are no modifications done.
- Fieldwork

### Data collection period

The data collection was extended over a period of 3 months. Each child took the Buteyko educational program over the course of 4 weeks, one session each week. This study was carried out through three consecutive phases: assessment, implementation, and evaluation phase.

1. The assessment phase

The interview with the children and parents was individual. During this phase, the informed consent was signed from the parent how to accept participation for 4 weeks after explaining the aim of the study, tool components, the session plan, and the steps of the Buteyko technique (BBT). Then, the assessment tools were completed.

Measuring PEFR was done in three steps:

1. Height measuring
  Each child stands with a straight back and bare feet facing the height measurement tape. This approximation was made in order to determine the predicted PEFR.
2. Determine the predicted PEFR
  The predicted PEFR was calculated by the following equation formula:
  [Predicted average PEF [L/min] = (Body height [cm] × 5.3)–433] [24].
3. Measuring the actual PEFR

The measuring steps according to the American Lung Association (2019) are as follows:

1. Each child was studied in the direct sitting posture.
2. The disposable mouthpiece was calibrated to the peak flow meter mouthpiece.
3. The pointer was shifted to zero.
4. The child was told to maintain (horizontally) the level of the peak flow meter and to keep his fingers away from the pointer.
5. The child was asked to take a deep breath and tightly close his lips around the plastic disposable mouthpiece.
6. The child was ordered to puff as much as he could.
7. The child was told to note that it is determined by the velocity of his puff.
8. The readings have been taken.
9. The pointer shifted to zero again.
10. This was replicated three times by each child and the highest reading was recorded.
11. During the study period, each child was examined for 4 sessions, one each week, to follow up the prognosis of child.

According to the following equation, the percentage of PEFR was formulated by dividing the measured PEFR over the expected PEFR and multiply it to 100:

$$[\mathrm{PEFR\%}\hspace{0.17em}=\hspace{0.17em}(\mathrm{measured PEFR}\backslash \mathrm{Predicted PEFR})\hspace{0.17em}\times \hspace{0.17em}100]$$

Evaluation procedure of control pause breathing test

According to the Buteyko Clinic International (2014), the CP steps are the following:

1. The child was instructed to sit and take a relaxed posture in a vertical chair, relax his shoulders, and lean his lower back against the back of the chair.
2. Until conducting CP, the child was told to avoid alternate breathing, the child was asked to take a small breath in (2 s) and a small breath out (3 s) and clutch the nose with empty lungs but not too long to clear on the “out” breath. To prevent air from escaping through the airways, clutching the nose is necessary.
3. The researcher estimates how many seconds the child can continue safely before they need to breathe in again.
4. The child was told to hold the breath until the first need to breathe in was felt. Release the nose then and naturally breathe through it smoothly.
5. The first intake of breath after the CP should be no higher than the breath before taking the measurement; the child should not hold the breath for too long as this could lead the child to take a major breath after measuring the CP.

2) The implementation phase

This phase is divided into two parts at each session (theoretical part and practical part) that took about 40 min for each child and mother.

### Theoretical part

During this part, a detailed explanation about the bronchial asthma, its causes, and effects was provided to the child and their parents. Then, the Buteyko technique, its benefits, and how to applying its steps were explained. The researcher used the educational booklet and story to simplify knowledge to parents, motivate children, and improve understanding. Finally, guidelines on lifestyle modification and the value of closing the mouth as possible at day and night were also discussed. Any question from the mother or child was answered, and any misunderstanding was corrected.

### Practical part

It included a demonstration of BBT until the child adequately understands its steps. In each session, the previous steps were revised with the child and their parents and adding the new steps till the fourth week of the Buteyko course. The implementation during the 4 weeks of exercise was as follows:

- Week one exercise
- Starting with nose breathing
- Relaxed breathing technique
- The control pause technique
- Advising of an hour of taping
- Daily nose-breathing walk technique
- Managing medication instruction
- Buteyko education about how to stop cough
- Week two exercise
- Extended pause technique
- Reduced breathing technique
- How to avoid colds and flu
- Night-time nose breathing instruction
- Week three and four
- Very reduced breathing technique
- Life style, diet, and sleep modification
  All this instruction and techniques are discussed in details in the booklet that is designed by the researcher and given to each mother with a Buteyko Steps Diary to follow her child at home in between sessions.

3) The evaluation phase

During this phase, the understanding of child and their parents was assessed. The child was asked to redemonstrate the Buteyko steps until the child well-practiced it. They were instructed to regularly do this technique at home. Parents were asked to help their children practice twelve repetitions of the Buteyko steps per day: four repetitions in the morning, four repetitions in the afternoon, and four repetitions in the evening. The parents were asked to record the number of steps done by their children in their Buteyko Steps Diary. Finally, they are answered for any question and asked to follow the modified life style.

### Statistical analysis

The collected data were organized, tabulated, and statistically analyzed using SPSS software (Statistical Package for the Social Sciences, version 20, SPSS Inc., Chicago, IL, USA). For quantitative data, the range, mean, standard deviation, and median were calculated. For qualitative data, which describes a categorical set of data by frequency, percentage, or proportion of each category, comparison between two groups and more was done using chi-square test (*χ*^2^). For comparison between means of two groups of non-parametric data of independent samples, *Z* value of Mann-Whitney test was used. For comparison between means of two related groups (pretest and posttest data) of parametric data, paired *t* test was used. For comparison between more than two means of parametric data, *F* value of ANOVA test was calculated, where Scheffe test was performed to compare between each two means if *F* value was significant. For comparison between more than two means of non-parametric data, Kruskal-Wallis (*χ*^2^) was calculated. A correlation between variables was evaluated using Pearson’s correlation coefficient (*r*). Significance was adopted at *p*<0.05 for interpretation of results of tests of significance.

## Discussion

Asthma is a major public health problem that will remain a challenge for the future if not treated correctly. Until now, this disease still receives minimal attention and care [25]. As regards the demographic data of the participants, the present study showed that more than half of them were females (Table 1). This was in disagreement with Priyalatha, Geetha, and Renuka [26] who conducted a study about the “Effectiveness of Buteyko breathing exercise (BBE) on respiratory outcome among children with bronchial asthma admitted in pediatric unit of Mgmcri,” which revealed that more than half of the studied children were males. In the same table, the current study showed that about two thirds of the participants aged from 6 to 10 years. This finding was in an agreement with the abovementioned study which reported that more than half of the studied children belonged to the age between 8 and 10 years. The researcher expected that as the current study sample was limited, the result cannot give an accurate distribution of age and sex.

**Table 1:** Percentage distribution of the studied children according to their demographic and clinical characteristics (*n* = 33)

Demographic data	The studied children (*n* = 33)	
	***n***	**%**
**Sex**		
Boys	16	48.5
Girls	17	51.5
**Age in years**		
6 ≤ 10	21	63.6
10–12	12	36.4
Mean ± SD	8.79 ± 1.69	
**Residence**		
Urban	14	42.4
Rural	19	57.6
**Educational level of caregiver**		
Illiterate	1	3.0
Low level	1	3.0
Moderate level	25	75.8
High level	6	18.2
**Diagnosis established since:**		
1- 3 Years	2	
More Than 3 Years	31	
**Family history of the disease:**		
Yes	22	
No	11	
**Factors that trigger asthma symptoms:**		
Colds and viruses	33	
Smoking around the child	31	
House dust mites	30	
Exercise	26	
Emotions	15	
Pollen	4	
Food allergies	3	
Other triggering factors ( perfumes, paints and flowers)	7	
**Frequency of asthma attacks per month:**		
1–3 time/month	22	
4–6 time/month	9	
More than 6 time/month	2	

It was illustrated from the same table that more than half of the participants came from rural areas. This finding was consistent with Mohammed et al. [25] who conducted a study about the “prevalence of bronchial asthma among school-aged children in the Elmaraghah center in Sohag governorate,” wherein they mentioned that children who live in rural areas had one and half time risk of developing asthma than those who live in urban areas. The researcher rationalizes this result as it may be due to poor sanitation, agricultural burning, and poor health awareness among rural population.

Regarding the clinical data, the current study revealed that most of children’s asthma is triggered by smoking and house dust (Table 1). This finding was in the same line with Varkey, and Yeshoda [27] who conducted a study on 60 children about the “effectiveness of breathing exercises in improving the breathing pattern of asthmatic children in a selected hospital” and reported that more than half of the studied sample was exposed to smoking and near to two thirds exposed to household dust. The current study finding revealed that approximately two thirds of the children have intermittent asthma attacks one to three times per month (Table 1) that was in congruent with Priyalatha et al. [26] who pointed that more than two-thirds of the studied children had attacks one to three times per month.

Regarding the family history of bronchial asthma, the current study pointed that about two thirds of studied children had family history of the disease (Table 1) that was supported by Priyalatha et al. [26] who indicated that more than half of the studied children had family history of bronchial asthma. This finding may be explained in the light of the fact that family history of allergy and poor sanitation were documented as risk factors associated with prevalence of asthma in Egypt [25]. So it is very important to provide and expand educational programs for parent to increase awareness about risk factors of asthma and preventive measures.

As regards the childhood asthma control test (C-ACT), the current study found that the mean of C-ACT pre-Buteyko assessment was improved progressively in the post-Buteyko application assessment (*p* = 0.0001) with a high mean percent of change of post than pretest which was clinically and statically high significant change (Table 2). This result was in harmony with Hepworth, Sinha, Saint, & Hawcutt [28], who conducted a study about “assessing the impact of breathing retraining on asthma symptoms and dysfunctional breathing in children”; they reported that overall the mean change in score of C-ACT was + 4.9, which was clinically and statistically significant. The researcher contributed this change to the reduction in the state of hyperventilation that associated with asthma symptoms, which resulting in a decrease of carbon dioxide level in the blood that contributes to bronchospasm and secretion accumulation.

**Table 2:** Childhood asthma control test (C-ACT) scores and its change among studied children after applying Buteyko breathing technique (*n* = 33)

Changes of C-ACT scores	The studied children pre- and post-test score after applying Buteyko breathing technique (*n* = 33)		Paired *t* test	*P*
	**Pre-test**	**Post-test**		
**C-ACT scores**				
Range	4.00–20.00	13.00–24.00	10.988	0.0001*
Mean ± SD	12.45 ± 4.12	19.57 ± 3.33		
Median	12.00	20.00		
**Change of scores post- than pre-test**				
Range	2.00–19.00			
Mean ± SD	7.12 ± 3.72			
**Percent of change of scores post- than pre-test:**				
Range	13.00%-380.00%			
Mean ± SD	81.45 ± 81.33			

^*^Statistically significant (*P* < 0.05)

Regarding the peak expiratory flow rate (PEFR) finding pre- and post-Buteyko technique application. The current study revealed that there was a significant increase in the mean at the 4th week finding than the first one, with a high significant mean percent of changes of post-Buteyko finding than the previous one (*p* = 0.0001) (Table 3). This finding came in accordance with Priyalatha et al. [26] who stated that there was a significant improvement in PEFR of the studied sample post-Buteyko implementation.

**Table 3:** Peak expiratory flow rate (PEFR) scores and its change among the studied children over follow-up after applying Buteyko breathing technique (*n* = 33)

Changes of peak expiratory flow rate (PEFR) (L/min.)	Follow-up per week (*n* = 33)			
	**1st week**	**2nd week**	**3rd week**	**4th week**
**PEFR (L/min.):**				
Range	90–250	110–280	100–295	120–320
Mean ± SD	157.48 ± 47.71	189.85 ± 44.94	201.82 ± 47.84	225.30 ± 50.53
Median	150.00	190.00	210.00	220
***F* value**	11.513			
***P***	0.0001*			
**Scheffe test** ***P***	1st vs 2nd, 3rd & 4th week, *P* = 0.061*, 0.004* & 0.0001*			
	2nd vs 3rd & 4th week, *P* = 0.793 & 0.032*			
	3rd vs 4th week, *P* = 0.268			
**Change of PEFR at 4th week than 1st week:**				
Range	0–173			
Mean ± SD	67.82 ± 39.83			
**percent of change of PEFR at 4th week than 1st week:**				
Range	0–101.80%			
Mean ± SD	78.46 ± 40.2			

^*^Statistically significant (*P* < 0.05)

In the same line, Rahi et al. [15] who conducted a study about the “Effect of Buteyko method on lung functions among asthmatic patients in Al-Najaf nity” also correspond to the current study PEFR finding. They suggested that the pre-test and post-test scores of lung function tests varied substantially and concluded that the Buteyko procedure is successful in enhancing the function of the lungs of patients. The researcher owes this to the improvement of airflow in and out from the lung associated with the BBT.

Concerning control pause test (CP), the current study presented a significant progressive improvement in the mean of the CP result between first, second, third, and fourth week (*p* = 0.0001) with a highly significant mean percent of changes at fourth week post-Buteyko application than the first one (Table 4). This finding was supported by Priyalatha et al. [26] whose finding showed that the breathing holding time (BHT) pre-test median score in the experimental group and control group was 6 and 4, and post-test median score was 16 and 7, respectively, which indicated that BBT was significantly effective in improving BHT. The researcher hypothesized this improvement in the post-test to the decrease of the body sensitivity to CO_2_ level in the blood after Buteyko training that consequently decrease hyperventilation and bronchospasm that trigger asthma symptoms.

**Table 4:** Control pause test (CP) findings among the studied children over follow-up after applying Buteyko breathing technique (*n* = 33)

Change of control pause test (CP)	Follow-up per week (*n* = 33)			
	**1st week**	**2nd week**	**3rd week**	**4th week**
**CP test (s) findings:**				
Range	7.00–21.00	10.00–25.00	10.00–44.00	14.00–46.00
Mean ± SD	12.70 ± 3.30	17.39 ± 3.80	23.79 ± 8.73	30.94 ± 9.61
Median	13.00	18.00	24.00	32.00
***F* value**	42.715			
***P***	0.0001*			
**Scheffe test**	1st vs 2nd, 3rd & 4th week, *p* = 0.062, 0.0001* & 0.0001*			
***P***	2nd vs 3rd & 4th week, *p* = 0.004* & 0.0001*			
	3rd vs 4th week, *p* = 0.001*			
**Change of CP at 4th week than 1st week**				
Range	6.00–36.00			
Mean ± SD	18.24 ± 8.56			
**Percent of change of CP at 4th week than the 1st week**				
Range	43.00–360.00%			
Mean ± SD	147.45% ± 74.22			

^*^Statistically significant (*p* < 0.05)

Regarding heart rate (HR), the current study revealed that there was a significant positive decrease in the heart rate mean over the 4 weeks of follow-up (*p* = 0.001) with a minus percent of change between first and fourth week of the Buteyko exercise (Table 5). This result was in harmony with Subramanian and Arora [29] who conducted a study to assess the hemodynamic changes following BBT training for 2 weeks in hypertensive subjects. Which pointed that there was a reduction in HR and blood pressure following 2 weeks of Buteyko training and supported the significant effect of BBT on diastolic blood pressure and HR. The researcher hypothesized that this can be attributed to the vasodilation due to the carbon dioxide retention that further leads to the lowering of peripheral resistance and hence the reduction in diastolic blood pressure.

**Table 5:** Heart rate (HR) findings among the studied children over follow-up after applying Buteyko breathing technique (*n* = 33)

Changes of HR	Follow-up of heart rate (HR) per week (*n* = 33)			
	**1st week**	**2nd week**	**3rd week**	**4th week**
**Heart rate (HR) (B/min)**				
Range	84–120	83–118	80–110	82–112
Mean ± SD	97.45 ± 11.75	94.36 ± 8.74	91.45 ± 7.81	89.06 ± 6.65
Median	95.00	93.00	90.00	88.00
***F* value**	5.447			
***P***	0.001*			
**Scheffe test** ***P***	1st vs 2nd, 3rd & 4th week, *p* = 0.580, 0.064 & 0.003*			
	2nd vs 3rd & 4th week, *p* = 0.628 & 0.127			
	3rd vs 4th week, *p* = 0.757			
**Change of HR at 4th week than the 1st week**				
Range	− 32.00:4.00			
Mean ± SD	− 8.39 ± 7.74			
**Percent of change of HR at 4th week than the 1st week**				
Range	− 26.60–5.10%			
Mean ± SD	− 8.02% ± 688			

^*^Statistically significant (*p* < 0.05)

The current study HR result was contradicted with Rai, Hembrom, & Sharma [30] who conducted a “study on immediate effect of the Buteyko breathing technique on cardio-respiratory parameters in young adults.” It was found that the heart rate in the pre-test was lower than that in the post-test that takes a contradicted direction with the current study. Despite this contradiction, the abovementioned study agreed with the current study on the effectiveness of the Buteyko technique as it observed a significant increase in forced expiratory volume after 5 min of BBT. From the researcher’s point of view, the current study post-test reduction in HR may come from the relaxation state that is learned during the 4 weeks of the Buteyko exercise.

Finally, the current study found that there was a strong positive correlation between C-ACT post-test, PEFR, and CP at fourth week in addition to a significant negative correlation with the HR. This result was illustrated in Table 6. This may be explained in the light of the fact that the dysfunctional breathing (DB) associated with asthma can contribute to a poor asthma control, and the BBT is directed to improve this DB and decrease hyperventilation. Consequently, the improvement of the child’s ability to control the asthma symptoms is revealed in C-ACT followed by the improvement in PEFR and CP tests, while the negative correlation with the HR may be resulted from the relaxation state that is learned from the Buteyko technique.

**Table 6:** Correlation of childhood asthma control test (C-ACT) scores post-test and different variables at the 4th week of follow-up among the studied children after applying Buteyko breathing technique (*n* = 33)

Variables	**Control pause test (CP) findings (*n* = 33)**			
	**Childhood asthma control test (C-ACT) scores post-test**	**Peak expiratory flow rate (PEFR)**	**Control pause test (CP) findings**	**Heart rate (HR)**
	***r*** ***P***	***r*** ***P***	***r*** ***P***	***r*** ***P***
**Age years**	0.006 0.975	0.529 0.002*	− 0.028 0.878	− 0.285 0.108
**Height**	− 0.033 0.855	0.666 0.0001*	− 0.032 0.862	0.345 0.049*
**Body weight**	− 0.213 0.233	0.390 0.025*	− 0.076 0.675	− 0.274 0.123
**Frequency of asthma attacks per month**	− 0.254 0.153	− 0.161 0.370	0.052 0.773	0.156 0.386
**C-ACT scores**	-	0.531 0.001*	0.709 0.0001*	− 0.526 0.002*
**Peak expiratory flow rate (PEFR)**	0.531 0.001*	-	0.551 0.001*	− 0.428 0.013*
**Control pause test (CP)**	0.709 0.0001*	0.551 0.001*	-	− 0.474 0.005*
**Heart rate (HR)**	− 0.526 0.002*	− 0.474 0.005*	− 0.428 0.013*	-

^*^Statistically significant (*p* < 0.05)*. r* = *correlation coefficient*

This opinion was supported by Mohamed et al. [13] who conducted a study on the “Buteyko breathing technique: a golden cure for asthma.” It was shown that there was a statistically significant increase in the regulation of everyday asthma, asthma incidence, and PEFR- in patients with a significant positive association between these parameters after administering the Buteyko breathing exercise for 1 month over pre-application.

### Study limitation

From the researcher’s point of view, the current study hypothesis was fulfilled by these results; the implementation of the Buteyko breathing technique may improve the control of asthma severity among school-age children. The study may be restricted by the low sample size, the provision of specialist facilities for the assessment, and treatment of bronchial asthma. In addition, some parents do not agree in the value of chest physical therapy as a helpful bronchial asthma treatment. Furthermore, the willingness of children to complete the entire curriculum over 4 weeks often ranges from child to child. Moreover, the children’s neurological and physiological status may affect the severity and frequency of bronchial asthma attacks that have an impact on the treatment. Finally, children may not adhere to the assigned Buteyko course at home, and their parents may not follow them.

## Conclusion

Based on the findings of the current study, it was concluded that the use of the Buteyko technique as an assistant to monitor asthma displayed a major improvement in asthma control among school-age children with bronchial asthma. This is reinforced by the outcomes of the study, which revealed a reduction in frequency of asthma-related symptoms, activity limitation that observed from the significant improvement in C-ACT post-Buteyko application test than the previous one. In addition, an improvement in the lung function tests after 4 weeks of the Buteyko method administration is observed in PEFR, CP, and HR finding.

### Recommendations

1. Further studies regarding the Buteyko method is to be conducted to evaluate its difference from other breathing techniques in controlling and managing asthma attacks and its effect on the community setting with using larger probability sample to achieve generalization of the results.
2. The study can be done by extending the duration of exercise to 6 months for more reinforcement of the result, better improvement, and reduction of symptoms
3. Application of BBT beside traditional treatment modalities recommended to be provided for children with asthma and training nurses caring for those children about BBT to be used in-patient care.

## Data Availability

The datasets used and/or analyzed during the current study are available from the corresponding author on reasonable request.

## Abbreviations

CAM: Complementary alternative medicine
BBT: Buteyko breathing technique
PEFR: Peak expiratory flow rate
C-ACT: Childhood asthma control test
CP: Control pause
FEV1: Forced expiratory volume in 1 s
